# The Highly Pure Neem Leaf Extract, SCNE, Inhibits Tumorigenesis in Oral Squamous Cell Carcinoma via Disruption of Pro-tumor Inflammatory Cytokines and Cell Signaling

**DOI:** 10.3389/fonc.2019.00890

**Published:** 2019-09-13

**Authors:** Jay Morris, Cara B. Gonzales, Jorge J. De La Chapa, April B. Cabang, Christos Fountzilas, Mandakini Patel, Stephanie Orozco, Michael J. Wargovich

**Affiliations:** ^1^Department of Molecular Medicine, Long School of Medicine, University of Texas Health Science Center San Antonio, San Antonio, TX, United States; ^2^Department of Comprehensive Dentistry, University of Texas Health Science Center San Antonio, San Antonio, TX, United States; ^3^Department of Medicine, GI Medical Oncology, Roswell Park Comprehensive Cancer Center, Buffalo, NY, United States

**Keywords:** neem, *Azadirachta indica*, oral squamous cell carcinoma, oral cancer, prevention

## Abstract

Oral squamous cell carcinoma (OSCC) is a deadly disease that comprises 60% of all head and neck squamous cell cancers. The leaves of the Neem tree (*Azadirachta indica*) have been used in traditional Ayurvedic medicine for centuries to treat numerous oral maladies and are known to have significant anti-inflammatory properties. We hypothesize that a highly pure super critical CO_2_ Neem leaf extract (SCNE) prevents initiation and progression of OSCC via downregulation of intra-tumor pro-inflammatory pathways, which promote tumorigenesis. Hence, we investigated the anticancer effects of SCNE using *in vitro* and *in vivo* platforms. OSCC cell lines (SCC4, Cal27, and HSC3) were treated with SCNE while inflammation, proliferation, and migration were analyzed over time. SCNE treatment significantly inhibited OSCC cell proliferation and migration and reduced MMP activity *in vitro*, suggesting its potential to inhibit tumor growth and metastasis. The preventive effects of SCNE in ectopic xenograft and 4NQO-1 (4-Nitroquinoline-1-oxide) carcinogen-induced mouse models of OSCC were also evaluated. Indeed, xenografted nude mice showed significant reduction of OSCC tumor volumes. Likewise, SCNE significantly reduced the incidence of tongue dysplasia in the 4NQO-1 OSCC initiation model. In both OSCC animal models, SCNE significantly depressed circulating pro-cancer inflammatory cytokines (host and tumor-secreted) including NFkB, COX2, IL-1, IL-6, TNFα, and IFNγ. In addition, we demonstrate that SCNE downregulates STAT3 and AKT expression and activity *in vitro*. We also demonstrate that the primary active component, nimbolide (NIM), has significant anticancer activity in established OSCC xenografts. Lastly, we show that SCNE induces an M1 phenotype in tumor associated macrophages (TAMS) *in vivo*. Taken together, these data strongly support SCNE as means of preventing OSCC via downregulation of pro-cancer inflammatory cascades and NIM as a potential new therapy for existing OSCC.

## Introduction

World-wide, the risk for developing oral squamous cell carcinoma (OSCC) is ever-increasing in parallel with the global rise in tobacco use, alcohol consumption, and HPV exposure (1). OSCC comprise 60% of all head and neck squamous cell carcinoma (HNSCC), which is the 6th most common cancer in the world and the 8th most common cancer in the United States (2). Notably, OSCC can arise anywhere in the oral cavity with the most common sites being the lateral borders of the tongue, the floor-of-the-mouth, and the base of the tongue (3). Human papilloma virus (HPV) is a known driver of oropharyngeal (tonsillar) cancers, but is rarely found in the oral cavity (3). Furthermore, HPV-driven oropharyngeal cancers respond very well to conventional treatments (4). In contrast, OSCC are rarely due to HPV infection (<5% of oral cancers are HPV positive) and are often resistant to standard therapies (5) Furthermore, 50% of all OSCC patients will have a recurrence of their disease within 3 years of treatment completion resulting in death within ~7 months of the recurrence; even with additional treatment (6). Therefore, HPV negative OSCC are the focus of this investigation.

Although conventional treatments (surgical excision, radiation, and platinum-based chemotherapies) have improved the 5-year survival rates for OSCC patients with early disease, patients with late-stage disease (stage III and IV) have a 5-year survival rate as low as 34% (1). Furthermore, these statistics have not changed in nearly 40 years. Hence, prevention of OSCC initiation and progression is critical to reducing the morbidity and mortality of this devastating disease.

*Azadirachta indica*, or Neem, belongs to a family of trees related to mahogany; Meliaceae (7). Neem is native to India, Myanmar, Bangladesh, Sri Lanka, Malaysia, and Pakistan and grows in tropical and semi-tropical regions around the world (7). The Neem tree is a source of highly active liminoid terpenoids, collectively known as azadiractoids which are shown to have anticancer activity (8). Previous studies of the anticancer potential of Neem in OSCC are limited to relatively impure ethanolic organic extracts from the Neem leaf evaluated in the hamster cheek pouch carcinogenesis model, where some activity is shown (9, 10). Murine models of stomach and skin cancers also demonstrate efficacy of the Neem leaf ethanolic extract (11). More recently, we demonstrate that a highly pure supercritical CO_2_ extract of the Neem leaf (SCNE) significantly reduced colon cancer tumor growth, inflammatory markers, and cell migration both *in vitro* and *in vivo* (12). Given that the Neem tree has been used for centuries to treat oral diseases, we evaluated its effects against OSCC as it relates to inflammation-driven tumorigenesis.

There are numerous inflammatory cytokines involved in OSCC development and progression, such as: IL-1β, IL-6, IL-8, IL-10, and tumor necrosis factor-alpha (TNFα) all of which are upregulated in patients with malignant OSCC (13–18). An environment of chronic inflammation yields IL-6, which promotes OSCC tumor growth and progression (19). For instance, exogenous IL-6 induces matrix metalloproteinase 2 (MMP2) and MMP9 in OSCC cell lines and thus may enable metastatic spread, and worsening survival rates (20). In addition, IL-6 regulates signal transducer activator of transcription-3 (STAT3) activation in HNSCC independent of EGFR signaling (13, 19). IL-8 is a known autocrine regulator of OSCC growth and contributes to cell motility (21). Lastly, salivary levels of IL-6, IL-8, IL-10, and TNFα are all increased in patients with malignant OSCC and thus are proposed to be discriminative biomarkers for oral cancer with IL-6 being associated with poor response to therapy and poor prognosis (14, 16, 18, 22–24). These studies strongly suggest that chronic inflammation potentiates the progression of OSCC.

In the current study we investigated the anticancer potential of a highly pure SCNE, in which the bioactive component, NIM, has been identified and all potential solute contaminants have been removed (25). SCNE was evaluated for antiproliferative, anti-inflammatory, and antimetastatic potential *in vitro* and *in vivo* using cell based assays, a 4-nitroquinoline 1-oxide (4NQO-1) carcinogen model of OSCC initiation, and three mouse xenograft models of human OSCC progression. Effects on circulating cytokines, inflammatory markers, and apoptotic markers are demonstrated; specifically dramatic downregulation of TNFα, IL-6, and downstream modulator STAT3 expression and activity are demonstrated. This inflammatory inhibition provides a potential mechanism for the preventive and therapeutic efficacy of SCNE against OSCC.

## Materials and Methods

### Reagents-Neem Extract

The supercritical CO_2_ Neem extract was provided by Nisarga Ltd., Sartara, India. Leaves from organically grown Neem trees under Good Agricultural Practices were processed with supercritical CO_2_ extraction technology and shipped to our laboratory. Supercritical extracts have the advantage of replacing organic solvents with excellent solvency. Such extraction results in no organic residues remain (26). We previously reported that NIM is present in the SCNE and was further evaluated as a bioactive in the current study (12). Stock solutions of 100 mg/ml in 100% DMSO were used *in vitro*. NIM was purchased from Biovision (#2356, San Francisco, CA, USA) and dissolved in 100% DMSO to a stock of 5 mg/ml (this calculates to 10.7 mM NIM stock). SCNE diets were manufactured by Envigo (Madison, WI, USA) to deliver 200 mg/kg SCNE—SCNE was dissolved in corn oil and homogenously mixed with the remaining diet ingredients and formed into pellets. Celecoxib (PZ0008, Sigma, St. Louis, MO, USA) was dissolved in 100% DMSO to a stock of 10 mg/ml (this calculates to a 26 mM Celecoxib stock).

### Human OSCC Cell Lines and MTT Assay

Cal27, HSC3, and SCC4 OSCC cell lines used in these studies were derived from primary tongue tumors and are HPV negative. HaCaT keratinocytes cells were used as controls. Cal27, SCC4, and HaCaT cells were obtained from ATCC (Manassas, VA) and HSC3 cells were kindly provided by Brian Schmidt's lab at NYU School of Denistry. All cell lines were validated by Genetica DNA Laboratories (Cincinnati, OH, USA) within 6 months prior to use. Cells were maintained in DMEM supplemented with 10% fetal bovine serum and 1% penicillin/streptomycin at 37°C with 5% CO_2_. For the SCC4 cells, hydrocortisone was provided at 400 ng/ml in the completed media. For all cell treatments we used SCNE (Nisarga Ltd.) applied at different concentrations (1–400 μg/ml) for 8, 24, or 48 h to 75% confluent cells. Doses to be tested bracketed the IC_50_ for each cell line. Final DMSO concentrations were maintained at 0.1% for all concentrations and test groups were compared to vehicle-treated controls (27). NIM was applied at different concentrations (1–100 μM) for 8, 24, or 48 h to 50% confluent cells. For the celecoxib treatments cells were treated at different concentrations (1–200 μg/ml) for 8, 24, or 48 h. Cells were cultured overnight in complete media, serum-starved for 24 h, and treated with vehicle, SCNE, NIM, or celecoxib as described above. Subsequently, 10 μl of 12 mM MTT (Life Technologies; Carlsbad, CA, USA) solution was added to each well, incubated for 4 h at 37°C, and neutralized with DMSO. Absorbance was measured at 540 nm and percent viability was calculated.

### Gelatinase Zymogram

Gelatinase zymography was performed in 10% SDS polyacrylamide gel in the presence of 0.1% gelatin under non-reducing conditions. OSCC cells were grown in 96 well plates. Culture media (200 μl) was collected from each well (pool of 3X) and concentrated to final volume 20 μl. Culture media (20 μl) were mixed with sample buffer and loaded for SDS-PAGE without boiling. Following electrophoresis the gels were washed twice in 1X Zymogram Renaturing Buffer containing Triton X-100 (Thermo Scientific, Franklin, MA, USA) for 1 h at room temperature (RT) to remove SDS. The gels were then incubated in 1X Zymogram Developing Buffer containing the substrate (Thermo Scientific, MA, USA) for 48 h at 37°C and stained with 0.5% Coomassie Blue R250 (Company) in 50% methanol and 10% glacial acetic acid for 60 min and then destained. Upon renaturation of the enzyme, gelatinases digest gelatin in the gel and give clear bands against an intensely stained background. Protein standards and 2% fetal bovine serum (positive control) were run concurrently and appropriate molecular weights were determined by plotting the relative mobilities of known proteins (28). Images were quantified using image J (NIH) and average ± SE from 2 replicates.

### Cell Migration Assay

SCC4, Cal27, and HSC3 cells were cultured in 96-well plates in complete growth medium. A scratch was performed using a WoundMaker and visualized using the IncuCyte ZOOM real time imaging system (Essen BioScience, MI, USA) (12, 29). Cells were treated with 20 or 60 μg/ml SCNE and 10 or 50 μM NIM and imaged at 3 h intervals for 120 h (SCC4), 72 h (Cal27), and 8 h (HSC3) to monitor cell migration and wound healing.

### Protein Expression

Cellular protein extracts were prepared and proteins quantified as described previously (12, 29–31). Briefly cells were washed twice with 1X PBS, collected by scraping and centrifuged at 4°C at 300 g for 6 min. The pellet was resuspended in 250 μl of Buffer A (10 mM Tris-HCl pH 7.8, 10 mM KCl, 1.5 mM MgCl_2_, 1 tab protease inhibitor and water) and incubated on ice for 10 min. The samples were homogenized at 15,000 rpm for 45 s on ice and then centrifuged at 4,600 g for 5 min at 4°C. The supernatent was removed and stored in −80°C as the cytosolic protein fraction. The collected pellet was resuspended in 100 μl of Buffer B (210 mM Tris-HCl pH 7.8, 420 mM KCl, 1.5 mM MgCl_2_, 20% glycerol, 1 tab of protease inhibitor and water) followed by gentle agitation at 4°C for 30 min and centrifugation at 10,000 g for 10 min at 4°C. The supernatant was collected and stored at −80°C as the nuclear protein fraction (29, 31). Cytosolic and nuclear protein fractions (50 μg)were separated by SDS-PAGE (12% gels) and transferred to PVDF membranes (Bio-Rad, Hercules, CA, USA). The membranes were probed with primary antibodies against nuclear factor kappa-B (NFkB) p65 (8242S), STAT3 (8768S) pSTAT3 (9131S), cycloxagenase 1 (COX1) (9896S), cyclooxagenase 2 (COX2) (12282S), EGFR (4267S), pEGFR (4404S), ERK1/2 (T202/Y204–9101S), AKT (9272S), pAKT (9271S) (Cell Signaling, Davers, MA, USA) followed by horseradish peroxidase-conjugated anti-rabbit (7074S; Cell Signaling). GAPDH (2118S; Cell Singaling) or Topo IIα (12286S; Cell Signaling) were used to ensure equal protein loading. The immunoreactive bands were visualized on ChemiDoc Touch (Bio-Rad, Hercules, CA, USA) using chemiluminescent substrate (Clarity ECL, Bio-Rad, Hercules, CA, USA). Images were quantified using image J (NIH) and average ± SE from 3 replicates.

### Animal Studies

All animal studies were approved by the University of Texas Health Science Center at San Antonio (UTHSCSA) Institutional Animal Care and Use Committee and followed the international guidelines on animal welfare in accordance with the National Institutes of Health guide for the care and use of laboratory animals. In addition, all animal studies comply with the ARRIVE guidelines and the 2013 American Veterinary Medical Association (AMVA) euthanasia guidelines. All mice were acclimated for 1 week prior to study initiation. An *n* = 10 per test group was used based on the following power analysis: assuming SCNE will cause a modest 30% decrease in OSCC tumor growth compared to control with 8 animals per group, two-sided testing, and alpha = 0.05, this study will achieve 85% power.

### OSCC Mouse Xenograft Models

Six week-old female athymic nude mice (Harlan, Indianapolis, IN, USA) were used in a laminar air-flow cabinet under pathogen-free conditions. They were provided with a 12 h light/dark schedule at controlled temperature and humidity with food and water *ad libitum*. Mice (*n* = 10) were injected subcutaneously in the right flank with 10 × 10^6^ SCC4, 6 × 10^6^ Cal27, or 3 × 10^6^ HSC3 cells in 0.2 ml of sterile PBS as previously described (12, 29). Mice were placed on AIN76A synthetic diet for 24 h following inoculation. Then the AIN76A diet supplemented with SCNE diet (200 mg/kg body weight) was provided to the SCNE treatment group while the control group remained on the standard AIN76A diet for the remainder of the study. For the HSC3 animal groups, NIM was administered by intraperitoneal (IP) injections for 5 consecutive days, starting at day 10 post tumor inoculation, at 5 or 20 mg NIM/kg body weight. Measurements were made every other day post injection and tabulated once the tumor reached 3 mm along the longest axis (SCC4, day 9; Cal27, day 5; HSC3 day 13). Tumor volumes were calculated by the elliptical formula: 1/2(Length × Width^2^) (32). Blood was drawn at termination and serum isolated for cytokine analysis.

### Immunofluorescence (IF) Analyses TAM Polarization in Cal27-Derived Tumors

Animals were anesthetized and perfused with phosphate-buffered saline (PBS) followed by paraformaldehyde (4%, w/v) in PBS. The tumor was removed intact and post fixed overnight in paraformaldehyde, cryoprotected in 30% sucrose in PBS for 24–48 h at 4°C, and then frozen in OCT freezing compound (Ted Pella). Serial cryosections were collected through the entire tissue (12 μM) and incubated with the rabbit polyclonal primary antibodies (diluted 1:50; anti-Arginase-1; Cell Signaling #93668 and anti-iNOS; BD Biosciences #610332) followed by fluorescent labeled secondary antibody (diluted 1:1000; anti-rabbit IgG (33). All samples were examined and images obtained using a Leica confocal microscope.

### CBA Carcinogen Induced Oral Cancer Initiation Model

This 12-week model induces dysplasia in 80% of mice at experimental conclusion. Briefly, 6 week-old female CBA mice (20 total) were placed on AIN76A diet or 200 mg/kg SCNE supplemented AIN76A diet and given 4NQO-1 (Sigma, St. Louis, MO, USA) at 100 μg/ml in their drinking water. The mice were kept on the 4NQO-1 water for 8 weeks, followed by 4 weeks of regular water. At 12 weeks, blood was drawn at termination for serum cytokine analysis and tongues excised and fixed in formalin for histological analysis.

### Immunohistochemistry

Formalin fixed tongue were paraffin embedded and sliced at 1 microns. Immunostaining was done following the previously published method (31) using the following antibodies (Abcam, Cambridge, MA, USA: PCNA ab18197; Ki-67 ab16667).

### Cytokine and Chemokine Assay

Serum cytokine/chemokine profile were taken at termination and stored at −80°C until analysis using the Bio-Plex Pro group 1 mouse cytokine 23-plex assay kit and analyzed with the Bio-Plex 200 Luminex-based multiplex analysis system (Bio-Rad, Hercules, CA).

### Statistical Analysis

Statistical analysis was performed using GraphPad Prism4 (San Diego, CA, USA). Cell viability and migration assays were analyzed by one-way ANOVA and Bonferroni's *post-hoc* test. Statistical analyses of tumor growth were made using analysis of variance with repeated measures with Bonferroni's *post-hoc* test. A *p*-value <0.05 was considered statistically significant.

## Results

### SCNE and NIM Inhibit Oral Squamous Cell Carcinoma Proliferation *in vitro*

Previous reports indicate slight anticancer activity of alcohol-derived Neem extracts, as well as inhibitory effects with NIM; one of several liminoids found in Neem leaf extracts (12, 34, 35). However, to date there are no reports investigating the anticancer potential of a solvent free, supercritical CO_2_ extract of Neem leaves containing both hydrophobic an hydrophilic constituents.

**Figure 1:**
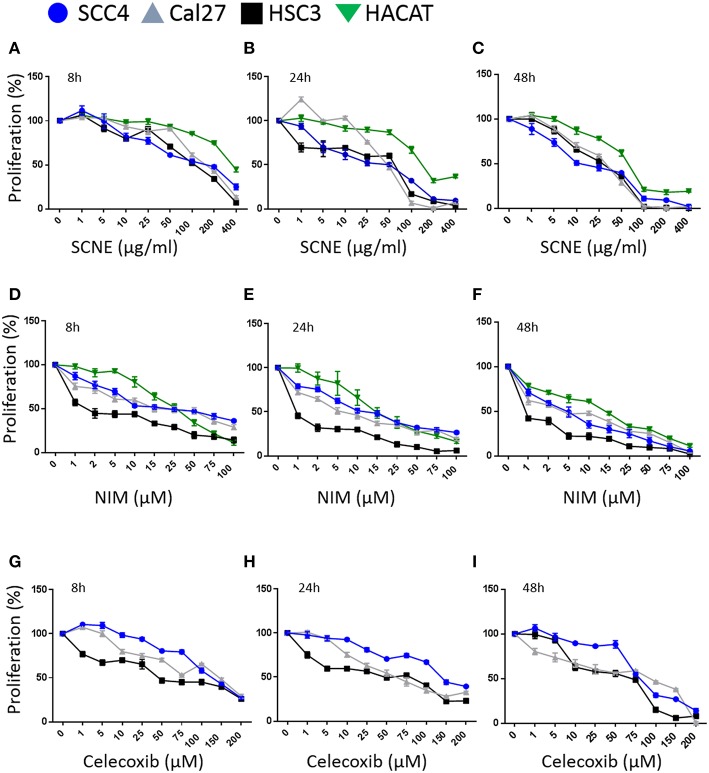
SCNE and NIM inhibit oral squamous cancer cell growth. **(A)** SCNE treatment (0–400 μg/ml) for 8 h, **(B)** 24 h, and **(C)** 48 h on SCC4, HSC3, and Cal27 OSCC cell lines. **(D)** NIM treatment (0–100 μM) for 8 h, **(E)** 24 h, and **(F)** 48 h on SCC4, HSC3, and Cal27 OSCC cell lines. **(G)** Celecoxib treatment (0–200 μM) for 8 h, **(H)** 24 h, and **(I)** 48 h on SCC4, HSC3, and Cal27 OSCC cell lines.

Utilizing SCNE and NIM, we treated three different OSCC human cell lines at increasing concentrations and three time points (8, 24, 48 h) to determine cytotoxic concentrations and the IC_50_ (Figures 1A–F). The SCNE reduced cell growth in a concentration and time dependent manner with IC_50s_ ranging from 100–200 μg/ml at 8 h to 50–75 μg/ml at 24 h, depending on the cell line tested (Figures 1A,B). The SCNE only showed modest toxicity, in a normal keratinocyte HaCaT cells, at high levels of SCNE (200–400 μg/ml at 8 and 24 h). Likewise NIM displayed IC_50s_ ranging from 10–25 μM at 8 h to 2–15 μM at 24 h, depending on the cell line tested (Figures 1D,E). However, HSC3 cells treated with NIM for 24 h were more sensitive with an IC_50_ of 1 μM, compared to 10 μM for SCC4 and 5 μM Cal27 cells (Figure 1E). In the normal HaCaT cells, NIM showed similar toxicity at higher concentrations (50 μM) to those measured in the OSCC cell lines. This indicates that SCNE, which contains NIM, is toxic to OSCC cells with less toxicity in normal keratinocytes; therefore SCNE may have more clinical therapeutic potential. In contrast, NIM, one of the bioactive components in SCNE, could have limited clinical potential due to similar measured toxicities in OSCC and normal keratinocytes.

We then compared SCNE and NIM cytotoxic effects to a standard non-steroidal anti-inflammatory, celecoxib (Figures 1G,H). At 8 and 24 h treatments the IC_50_ for celecoxib was 50 μM for Cal27 and HSC3, while the SCC4 IC_50_ was 150 μM. The strongest effects were measured at 48 h with an IC_50_ range of 10–25 μg/ml SCNE in all three cell lines tested (Figures 1C,F,I). For NIM an IC_50_ below 1 μM was observed for HSC3 and 5 μM for SCC4 and Cal27 cell lines. In the celecoxib treated Cal27 and HSC3 the IC_50_ was 25 μM, while the SCC4 had a modest decrease to 75 μM. These results show that SCNE and NIM have greater cytotoxic effects on OSCC cell lines than standard NSAIDs. From this data we chose 20 and 60 μg/ml SCNE and 10 and 50 μM NIM, along with 8 and 24 h time points to further study the mechanisms-of-action.

### SCNE and NIM Down Regulate Inflammatory Mediators and STAT3, AKT, and ERK1/2 Signaling

**Figure 2:**
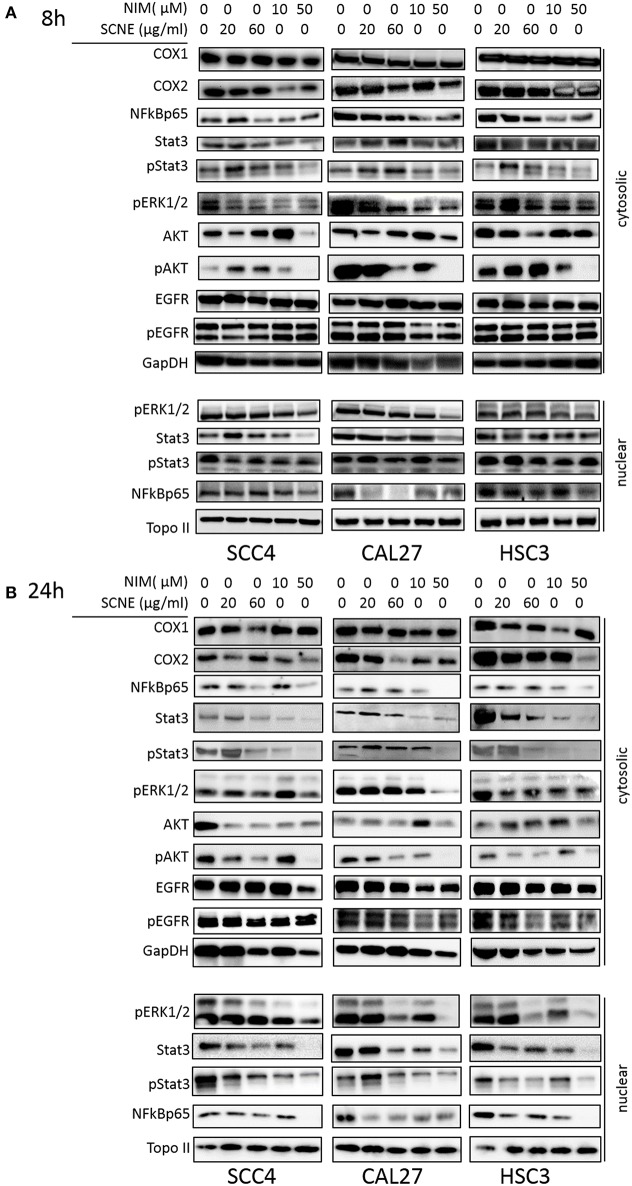
SCNE and NIM down regulate inflammatory mediators. SCC4, Cal27, and HSC3 cells were treated with 20 μg/ml, 60 μg/ml SCNE, or 10 μM, 50 μM NIM for **(A)** 8 h or **(B)** 24 h Cytosolic protein fractions were analyzed for COX1, COX2, NFkBp65, STAT3, pSTAT3, EGFR, pEGFR, pERK1/2, AKT, and pAKT. Nuclear protein fractions were analyzed for pERK1/2, STAT3, pSTAT3, and NFkBp65. GapDH and Topo-IIα were used as loading controls.

To elucidate the mechanism(s)-of-action of SCNE and NIM we treated three OSCC cell lines with SCNE (20 and 60 μg/ml), NIM (10 and 50 μM), at two time points 8 and 24 h, then analyzed cytosolic and nuclear protein fractions (Figure 2) and quantified with Image J (Supplemental Figures 2, 3). Reports have shown that inflammatory markers, such as NFkB, cyclooxygenases, as well as cellular proliferators STAT3, AKT, and ERK1/2 are elevated in OSCC (13, 15, 23). Treatment with SCNE or NIM moderately decreased COX2 expression at 8 h in SCC4 cells with little effect on Cal27 cells (Figure 2A and Supplemental Figures 2A,B). NIM significantly reduced COX2 expression in HSC3 cells at 8 h (*p* < 0.01; Figure 2A and Supplemental Figure 2C). By 24 h SCNE and/or NIM demonstrated downregulation of COX2 expression in all cell lines with significant downregulation (*p* < 0.0001) in HSC3 cells (Figure 2B and Supplemental Figure 3). There was little effect on levels of COX1 protein in all cell lines and time points (Figures 2A,B and Supplemental Figures 2, 3). A modest decrease of cytosolic NFkBp65 was demonstrated using higher concentrations of SCNE and/or NIM in all cell lines tested, with the strongest downregulation at 24 h (Figure 2B and Supplemental Figure 3). However, nuclear NFkBp65 was significantly reduced in all three cell lines at 24 h. Both SCNE and NIM downregulated cytosolic pSTAT3, pAKT, and pERK1/2 at 24 h in all three cell lines (Figure 2B and Supplemental Figure 3) with only modest downregulation observed at 8 h (Figure 2A). Nuclear pSTAT3 and pERK1/2 were significantly downregulated in all OSCC cell lines at 24 h (Supplemental Figure 3). Significant decreases in EGFR and pEGFR were observed in cytosolic and nuclear fractions at 24 h for Cal27 and HSC3 cells; with no change in SCC4 cells (Figure 2B and Supplemental Figure 3). These results confirm the anti-inflammatory and antiproliferative effects of SCNE and NIM in OSCC.

### SCNE and NIM Inhibit *in vitro* Cell Migration

**Figure 3:**
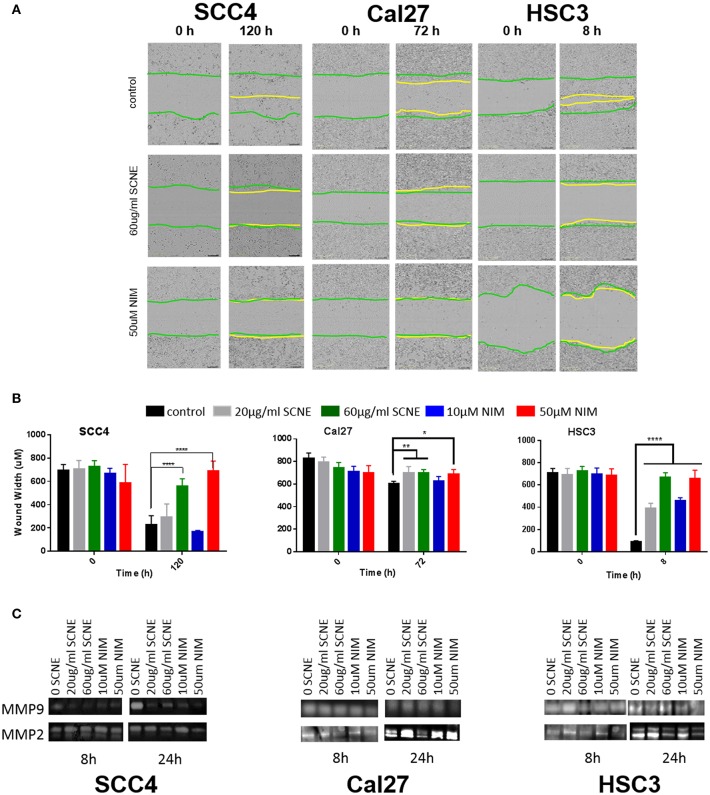
SCNE and NIM inhibit *in vitro* cell migration. **(A)** Wound-healing assay showing 60 μg/ml SCNE and 50 μM NIM inhibits cell migration in SCC4 (120 h), Cal27 (72 h), and HSC3 (8 h). Green lines represent initial wound front, yellow is wound front after respective treatment time. **(B)** Average wound width in SCC4, Cal27, and HSC3 is significantly reduced (*n* = 6, *p < 0.05, **p < 0.01, ****p < 0.001) with SCNE and NIM treatments. **(C)** Gelatinase zymograms showing MMP2 and MMP9 activity from SCC4, Cal27, and HSC3 treated cells.

Our *in vitro* results suggest that the cytotoxic effect on OSCC by SCNE and NIM (its prominent liminoid) act in part, through downregulation of inflammatory mediators and cellular signaling that promotes proliferation. To better understand the anticancer potential of SCNE and NIM, we assessed proxy assays for antimetastatic effects *in vitro*. Utilizing a wound healing assay found that both SCNE and NIM significantly reduce cell migration in all cell lines tested (Figures 3A,B). The highly-mobile cell line HSC3 closed the wound (90%) in 8 h. However, treatment with SCNE and NIM significantly inhibited this wound closure resulting in a mere 10% closure in the same time course (*p* < 0.001). In the low-mobile SCC4 cell line, which requires 120 h to close the wound 100%, SCNE and NIM completely halted cell migration across the wound (Figures 3A,B; *p* < 0.001). Cal27 cells required 72 h to measure any discernable closure of the wound; however SCNE and NIM did inhibit this nominal cell migration compared to the untreated group (*p* < 0.05).

Given these strong results barring cellular migration, we assessed the effects of SCNE and NIM on two matrix metalloproteinase proteins, MMP2 and MMP9. In the highly mobile HSC3 OSCC cell line, 24 h treatment with SCNE (60 μg/ml) and NIM (50 μM) significantly reduced MMP2 and MMP9 activity by at least 50% (Figure 3C and Supplemental Figure 1). In the SCC4 cell line, MMP9 was drastically reduced by SCNE and NIM at all concentrations tested, with reductions in MMP2 that were not significantly significant (Figure 3C and Supplemental Figure 1). Cal27 cells had significant reductions in MMP2 activity with SCNE (60 μg/ml) treatment and MMP9 activity with NIM (50 μM) treatment at the 24 h time point. Conversely, the low concentration treatments with SCNE and NIM induced a small, but insignificant increase in both MMP2 and MMP9 at 24 h. Given that Cal27 cells have low migration capabilities, changes in MMP2 and MMP9 most likely do not mediate their metastatic potential. However in the low-mobile SCC4 and highly-mobile HSC3 cells, both SCNE (60 μg/ml) and NIM (50 μM) dramatically inhibited cell migration and significantly reduced MMP2 and MMP9 activity (Supplemental Figure 1). Taken together, these *in vitro* results suggest that SCNE and NIM may mediate OSCC metastases via inhibition of migration and downregulation of MMP2 and MMP9.

### SCNE and NIM Inhibits OSCC Derived Tumor Growth in Mice

**Figure 4:**
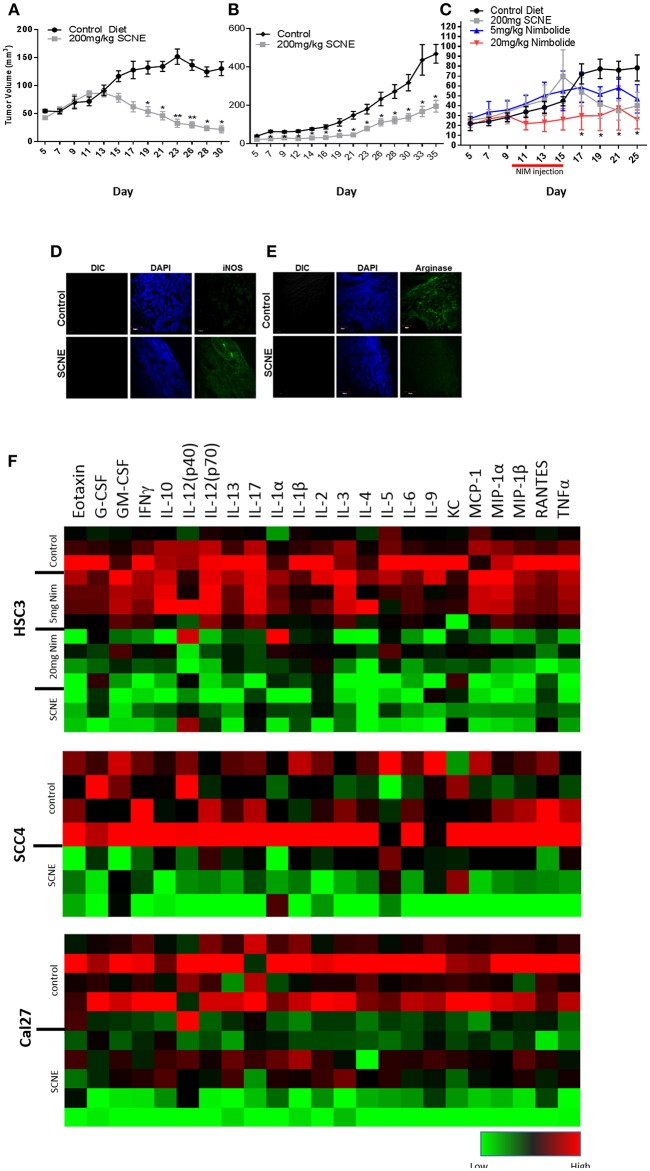
SCNE and NIM inhibits OSCC derived tumor growth in mice and reduces inflammatory cytokines. **(A–C)** SCNE supplementation (200 mg/kg) of the AIN76A diet significantly inhibited tumor growth in OSCC mouse xenografted models; [**(A)**: SCC4−30 day treatment yielded−81.12% reduction in tumor volumes; **(B)**: Cal27−35 day treatment yielded−49.00% reduction in tumor volume; and **(C)**: HSC3−25 day treatment yielded−48.81% reduction in tumor volumes] (*p < 0.005, **p < 0.001). **(C)** NIM treatment (20 mg/kg IP) significantly reduced HSC3 tumor volumes (66%; *p < 0.05) in mice after 25 days. **(D,E)** SCNE produces M1 phenotype in TAMs. **(F)** Inflammatory cytokine levels from SCC4, Cal27, and HSC3 xenografted mice are reduced when fed SCNE (200 mg/kg) in diet and IP injected NIM.

To corroborate the results from the *in vitro* studies, we used the same OSCC cell lines to generate three xenograft mouse models (Figure 4) and evaluated the antitumor efficacy of dietary SCNE. We used SCNE (200 mg/kg) incorporated into the AIN76A diet to deliver the therapeutic neem extract and the synthetic AIN76A diet as control. Briefly, mice were inoculated with OSCC cells and placed on the SCNE or control diets the following day. Tumor incidence and volumes were followed over time. At experimental conclusion the SCNE supplemented diet significantly reduced SCC4 tumor volumes (81%, Figure 4A) and Cal27 (49%, Figure 4B) tumor volumes compared to the untreated controls (Figures 4A,B; *p* < 0.05). SCNE also reduced HSC3 tumor volume (49%, Figure 4C), however due to high variance statistical significance was not achieved.

As a proof-of-principle study, we also tested the therapeutic effects of NIM in the HSC3 xenograft model by allowing tumors to establish for 10 days prior to initiating NIM treatments. Nim (5 or 20 mg) was administered by intraperitoneal injection (IP) for 5 consecutive days. Tumor growth was followed for 25 days. The 20 mg/kg NIM treatment significantly (*p* < 0.05) reduced tumor volume (69%) while the 5 mg/kg showed a moderate reduction (40%) in volume (Figure 4C). In all SCNE diet and NIM studies, body weight, motor function, activity levels, and food consumption were comparable between experimental arms (data not shown). These data affirm both SCNE and NIM have profound antitumor activity *in vivo*.

### SCNE and NIM Reduce Serum Level Inflammatory Cytokines in Xenografted Mice

We analyzed the serum from the foregoing experiment to investigate the effects of SCNE on the circulating inflammatory cytokine population (Table 1, Figure 4F). The extract-treated mice evidenced reduced IL-1β, TNFα, IFNγ, and IL-6 serum levels. For IL-1α, there was a moderate reduction in all three OSCC cell line xenografted mice. A similar pattern was observed for IL-10 but the stronger reduction occurred in the HSC3 tumor-bearing mice. We examined many other cytokines from these animals (Table 1, Figure 4F) and overall SCNE treatment produced a unique profile exhibiting a dramatic reduction of inflammatory cytokines (green) compared to control (black and red). Combined, our data demonstrates that SCNE reduces tumor burden and downregulates circulating levels of pro-tumor inflammatory cytokines.

**Table 1:** SCNE reduces serum level inflammatory cytokines in xenografted and carcinogen induced mouse models of OSCC.

	**Xenograft**									**Carcinogen**		
**Animal study**	**Cal27**			**HSC3**			**SCC4**			**4NQO-1/CBA**		
**Cytokine (pg/ml)**	**Con**	**SCNE**	**%**	**Con**	**SCNE**	**%**	**Con**	**SCNE**	**%**	**Con**	**SCNE**	**%**
IL-10	107.72	77.76	−27.8	153.46	42.59^*^	−72.2	291.08	152.74	−47.5	96.63	65.27^*^	−32.5
IL-1α	25.40	19.84	−21.9	45.13	28.63	−36.6	62.02	51.60	−16.8	7.69	7.23	−6.0
IL-1β	32.93	21.47	−34.8	1660.65	145.25^*^	−91.3	512.95	360.32	−29.8	380.08	282.22^**^	−25.8
Il-6	41.00	23.78	−42.0	170.30	22.21^*^	−87.0	67.19	45.28	−32.6	22.26	17.71	−20.4
TNF α	600.39	333.41^*^	−44.5	3089.10	468.30^*^	−84.8	2180.25	1366.18	−37.3	599.96	467.77^*^	−22.0
IFNγ	110.68	55.98^*^	−49.4	425.41	64.03^*^	−85.0	277.86	183.63^*^	−33.9	79.49	47.94^*^	−37.0

* p < 0.05;

** *p < 0.01. The values highlighted in green are statistically significant*.

Although athymic nude mice lack T-cells, they do produce B-cells. Therefore, in order to gain insight into SCNE potential effects on host TAM polarization, we analyzed markers of M1 (iNOS) and M2 (Arginase 1) phenotypes in Cal27-derived tumors from mice on control and SCNE diets using IF. Indeed IF analyses confirmed that dietary SCNE downregulates M2 TAMs (pro-tumor) and upregulates M1 TAMs (antitumor) *in vivo* (Figures 4D,E). This is consistent with other reports using Neem leaf glycoprotein (NLGP) to shift human M2 macrophage polarization (in response to human laryngeal tumor cell lysates) back to an M1 phenotype *in vitro* (36).

### SCNE Inhibits Tumor Initiation in 4NQO-1 Mouse Model of OSCC

**Figure 5:**
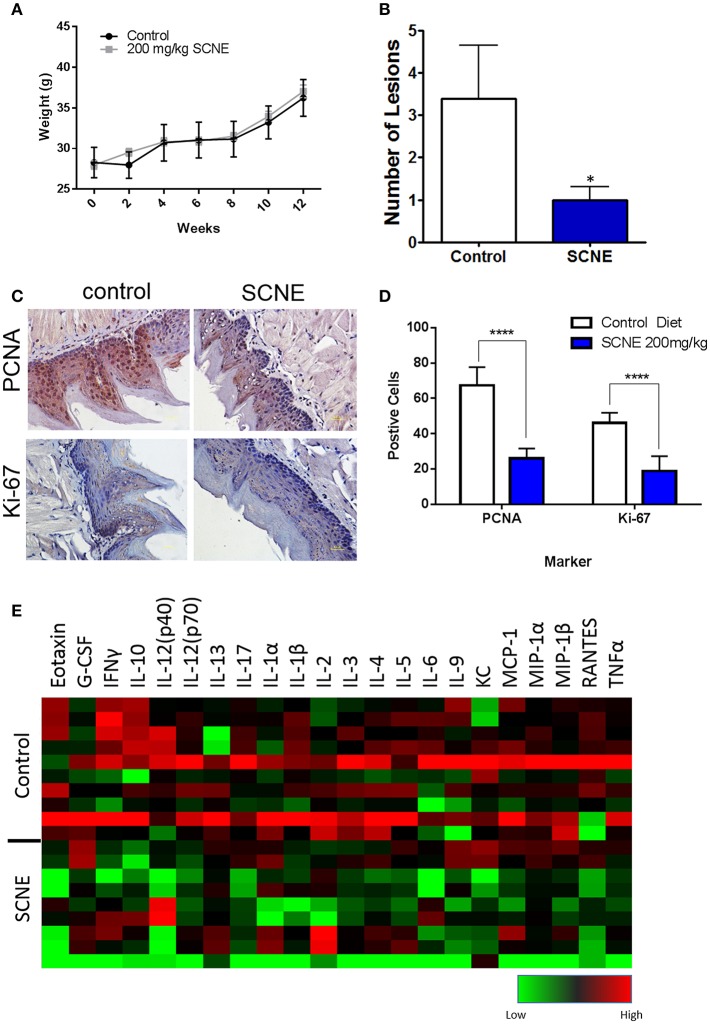
SCNE inhibits tumor growth in 4NQO-1 mouse model of OSCC. **(A)** CBA mice showed no difference in weight gain on 200 m/kg SCNE supplemented diet over the 12 week study. **(B)** SCNE diet reduced tongue lesions 5-fold (*p < 0.05) and tongue carcinomas compared to control diet. **(C)** SCNE diet reduced levels of proliferating markers PCNA and Ki-67 in mouse tongues. **(D)** SCNE reduced the number of cells expressing PCNA and Ki-67 (three regions from three separate tissues, ****p < 0.0001). **(E)** Inflammatory cytokine levels from 4NQO-1 mouse model are reduced when fed SCNE (200 mg/kg) in diet.

To further validate the *in vivo* anticancer potential of SCNE, we utilized a 12-week 4NQO-1 OSCC initiation model in the CBA mouse strain. The 4NQO-1 (100 μg/ml) was administered in the drinking water for 8 weeks and then regular water replaced it for another 4 weeks. The control mice were fed *ad libitum* AIN76A control diet while the treatment group was fed SCNE (200 mg/kg body weight) supplemented AIN76A diet for the entire 12 week study. To assess the palatability of the diet, we weighed the SCNE treated mice every 2 weeks and found no difference in their weight compared to mice on the control diet (Figure 5A). At termination we examined the tongues histologically for any dysplasia and/or tumors. SCNE diet significantly reduced the number of oral lesions compared to animals on control diet (Figure 5B; *p* < 0.05). We also characterized expression levels of proliferative markers in the tongues by immunohistochemistry and found SCNE reduced PCNA and Ki-67 protein levels (Figure 5C). The quantitated numbers of positive cells from three regions from three separate images show a significant reduction in PCNA and Ki-67 expressing cells due to SCNE treatment (Figure 5D; *p* < 0.0001). These results corroborate our xenograft results further demonstrating that SCNE inhibits tumorigenesis *in vivo*.

### SCNE Reduces Serum Inflammatory Cytokines in a 4-NQO-1 Carcinogen Induced Mouse Model of OSCC

In addition to the pathobiology of the 4NQO-1 treated mice, we examined the serum circulating cytokine inflammatory population. SCNE significantly reduce IFNγ, IL-1β, and TNFα levels in these animals (Table 1; ^*^p < 0.05, ^**^p < 0.01). Circulating IL-6 and IL-1α levels were reduced after 12 weeks of SCNE diet consumption (30 and 25%, respectively). We examined additional cytokines from these animals (Figure 5E) in which SCNE treatment yielded a dramatic reduction in circulating pro-cancer inflammatory cytokines (green) compared to control mice (black and red). This pattern paralleled dramatic reductions in circulating cytokines demonstrated in the xenograft animal studies further validating the anticancer and anti-inflammatory effects of SCNE.

## Discussion

This study establishes the significant anticancer effects of SCNE in OSCC through downregulation of key mediators of tumor proliferation and pro-cancer inflammation. SCNE also significantly reduced cell migration, suggesting the potential to inhibit metastasis. Numerous studies evaluating inflammatory diseases of the oral cavity establish an increased risk of oral premalignant lesions and OSCC with chronic periodontal disease and lichen planus (37–43). Cancer-related inflammation (CRI) stimulates a pro-growth tumor microenvironment by producing growth factors, chemokines, chemokine receptors, and MMPs (44–48). Important molecular events related to CRI pathways provide targetable modification of transcription factors (e.g., NFkB) and STAT3, cytokines, and assorted chemokines (17, 49–52). COX2 expression is also significantly increased in OSCC tissues and associated with advanced clinical staging (53). Our results reveal, for the first time, a strong impact of SCNE on the inflammatory signaling cascade via downregulation of NFkB, STAT3, and COX2 expression and/or activity in OSCC cell lines.

We further demonstrate robust therapeutic and preventive effects of SCNE utilizing two unique animal models of OSCC; xenograft models evaluated therapeutic effects on tumor growth and 4NQO-1 carcinogen-induced model evaluated prevention of tongue lesions (dysplasia and OSCC). All three OSCC xenograft models showed significant reductions in tumor growth. The SCNE diet also significantly reduced early tongue lesions in the 4NQO-1 mouse model.

Similar to human studies, tumors from 4NQO-1 treated mice have increased TNFα and IL-1β levels compared to healthy mice (54). Impressively, the SCNE dramatically depressed key circulating pro-cancer inflammatory cytokines (IFNγ, TNFα, IL-6, IL-1α, and IL-1β) in all of the xenograft and 4NQO-1 mouse models tested. IFNγ is an important cytokine produced in several immune response cells such as natural killer, natural killer T cells, and activated T cells (55). This process can be beneficial in adaptive antitumor immune response (56) but can also be immunosuppressive by upregulation of molecules such as IDO1 in the tumor microenvironment (57) or through feedback inhibition which may reduce antitumor activity (58). While we observed strong reduction in IFNγ we also measured substantial reduction in tumor volumes in our xenograft studies (immuno-suppressed) and reduction in lesions in our carcinogen induced model of OSCC (immuno-competent); suggesting SCNE promotes an antitumor phenotype that is not dependent upon T-cell activity, but may be enhanced by a fully competent immune status.

Notably, we demonstrate for the first time that dietary SCNE shifts M2 TAMs to an M1 phenotype *in vivo*. Previous studies using a NLGP to assess macrophage polarization demonstrated downregulation of STAT3 activity in human macrophages and a shift to an M1 phenotype *in vitro* (36). Given that SCNE mediates STAT3 activity in OSCC, it is plausible that SCNE also mediates STAT3 activity in TAMs. Additional studies are underway to fully assess SCNE-induced changes in immune cell populations using immunocompetent mouse OSCC models.

Currently, the OSCC incidence is on the rise. Moreover, standard first-line therapies have little efficacy against advanced disease (59). Sadly, ~60% of patients are diagnosed with late-stage disease that is refractive to conventional therapies; consequently, 66% of those patients (with advanced disease) will die within 5 years (2). Even worse, 50% of all OSCC patients will experience recurrent disease within three years of definitive treatment resulting in death within seven months even with additional treatment (60). These statistics are due to the lack of efficacious therapies for advanced and recurrent disease. To improve this scenario, new avenues to treatment with novel agents and combination therapies could potentially overcome this issue. One study showed the potential of combining the targeting the PI3K/AKT/mTOR pathway using a PI3k/mTOR inhibitor with radiation; it was found that this modality improved cell cycle arrest and decreased radio-resistance in OSCC (61). COX2 expression is shown to be elevated in OSCC and contributes to radio-resistance (62). Given SCNE anti-inflammatory effects and reduction in NFkB/STAT3 axis, there is potential for future studies to measure the synergistic effects of SCNE in combination with standard platinum-based chemotherapies and radiotherapy to treat OSCC and reduce recurrence rates and mortality.

In conclusion, we examined the chemopreventive effects of SCNE derived from a natural source with ethnopharmacological properties, on OSCC tumorigenesis. We observed both *in vitro* and *in vivo* oral cancer model systems a marked decrease in OSCC proliferation, cell signaling (STAT3, AKT, and ERK1/2), and circulating pro-cancer cytokines (tumor-secreted and host). With the dearth of clinical treatment options for OSCC frontline and second-line therapies, this natural extract shows potential as a prevention agent in a standalone regime or in adjuvant combination with standard frontline therapies to improve patient outcomes and/or resistant recurrent tumors in relapsed patients. The latter notion awaits Neem-based clinical trials.

## Data Availability

The datasets for this study will not be made publicly available because this article does not contain any datasets.

## Ethics Statement

All animal studies were approved by the University of Texas Health Science Center at San Antonio (UTHSCSA) Institutional Animal Care and Use Committee and followed the international guidelines on animal welfare in accordance with the National Institutes of Health guide for the care and use of laboratory animals. In addition, all animal studies comply with the ARRIVE guidelines and the 2013 American Veterinary Medical Association (AMVA) euthanasia guidelines. All mice were acclimated for one week prior to study initiation.

## Author Contributions

JM, CG, and MW contributed to writing the manuscript, data analysis, experimental design, and data interpretation. JM, JD, AC, SO, and MP all conducted animals experiments, measurements, and data analysis. JM and MP completed the zymogram assays. JM and AC conducted the scratch assays and data analysis. CF did all the IHC analysis and data interpretation.

### Conflict of Interest Statement

The authors declare that the research was conducted in the absence of any commercial or financial relationships that could be construed as a potential conflict of interest.

## Supplementary Material

Supplemental Figure 1 Quantification of Zymogram analysis blots using image J. Means ± SE (*n* = 2), ^*^*p* < 0.05, ^**^*p* < 0.01, ^***^*p* < 0.001.

Supplemental Figure 2 Quantification of Western blots from 8 h treatments using image J. Means ± SE (*n* = 3), ^*^*p* < 0.05, ^**^*p* < 0.01, ^***^*p* < 0.001. **(A)** SCC4, **(B)** Cal27, **(C)** HSC3.

Supplemental Figure 3 Quantification of Western blots from 24 h treatments using image J. Means ± SE (*n* = 3), ^*^*p* < 0.05, ^**^*p* < 0.01, ^***^*p* < 0.001, ^****^*p* < 0.0001. **(A)** SCC4, **(B)** Cal27, **(C)** HSC3.

## Abbreviations

OSCC: Oral Squamous Cell Carcinoma.
